# Recurrent ischaemic strokes as a first presentation of Libman–Sacks endocarditis with an atypically massive mitral vegetation resulting in severe valvular regurgitation: a case report

**DOI:** 10.1093/ehjcr/ytaf647

**Published:** 2025-12-11

**Authors:** Pok-Tin Tang, Marco Spartera, Badrinathan Chandrasekaran, Jan Lukas Robertus, Steve Ramcharitar

**Affiliations:** Division of Cardiovascular Medicine, Radcliffe Department of Medicine, University of Oxford, Level 0 John Radcliffe Hospital, Headley Way, Headington, Oxford OX3 9DU, UK; Wiltshire Cardiac Centre, Great Western Hospital, Great Western Hospitals NHS Foundation Trust, Marlborough Road, Swindon SN3 6BB, UK; Division of Cardiovascular Medicine, Radcliffe Department of Medicine, University of Oxford, Level 0 John Radcliffe Hospital, Headley Way, Headington, Oxford OX3 9DU, UK; Wiltshire Cardiac Centre, Great Western Hospital, Great Western Hospitals NHS Foundation Trust, Marlborough Road, Swindon SN3 6BB, UK; Wiltshire Cardiac Centre, Great Western Hospital, Great Western Hospitals NHS Foundation Trust, Marlborough Road, Swindon SN3 6BB, UK; Department of Histopathology, Royal Brompton and Harefield Hospitals, Guy’s and St Thomas’ NHS Trust, Hill End Road, Harefield, London UB9 6JH, UK; Wiltshire Cardiac Centre, Great Western Hospital, Great Western Hospitals NHS Foundation Trust, Marlborough Road, Swindon SN3 6BB, UK

**Keywords:** Non-bacterial thrombotic endocarditis, Libman–Sacks endocarditis, Systematic lupus erythematosus, Ischaemic stroke, Mitral valve replacement, Case report

## Abstract

**Background:**

Libman–Sacks endocarditis is a form of non-bacterial thrombotic endocarditis, associated with autoimmune conditions such as systemic lupus erythematosus and antiphospholipid syndrome (APLS). Vegetations are usually small and are managed with immunosuppression and anticoagulation.

**Case summary:**

A 50-year-old female presented to her hospital with left leg weakness, with imaging showing a right parietal stroke and an old occipital lobe stroke. Inpatient transthoracic echocardiography showed a large mitral valve (MV) vegetation with moderate-to-severe mitral regurgitation (MR). She self-discharged against medical advice before further workup could be completed and was lost to follow-up until persuaded to have an outpatient transoesophageal echocardiogram, which showed severe MR with a large (2 cm × 3 cm) mass attached to the posterior MV leaflet. Blood cultures were negative. Review of previous blood tests showed a triple-positive APLS panel, which was positive on repeat testing. She underwent successful mechanical MV replacement. Valve histology was consistent with Libman–Sacks endocarditis. Warfarin therapy was continued, complicated by subdural haematoma (successfully treated), but with no further thrombo-embolic events. Subsequent anti-nuclear antigen testing was positive, and hydroxychloroquine was commenced. Transoesophageal echocardiography 1 year later showed a well-functioning MV prosthesis.

**Discussion:**

The management of young individuals with ischaemic stroke should include attention to atypical causes. Libman–Sacks endocarditis is usually associated with small vegetations and high thrombotic risk, usually managed medically with anticoagulation and treatment of underlying conditions. Our case was atypical, with the presence of a large vegetation causing significant valvular dysfunction, but it demonstrates that replacement with mechanical prostheses can be a feasible management strategy.

Learning points

- Work-up for ischaemic stroke in young patients requires consideration of aetiologies beyond traditional cardiovascular risk factors.
- Libman–Sacks endocarditis usually manifests as small vegetations managed with anticoagulation and immunosuppression, but rarely presents as large vegetations causing significant valvular dysfunction.
- Surgical valve replacement for Libman–Sacks endocarditis may be indicated where the vegetations are large enough to cause significant valvular dysfunction.

## Introduction

Infective endocarditis is the most common cause of valvular vegetations, and the usual priority differential diagnosis when vegetations are seen on imaging, given the potential for rapid clinical progression with haemodynamic deterioration and thromboembolism. However, non-infective causes, such as non-bacterial thrombotic endocarditis (NBTE), though uncommon, are important to recognize, as treatment is directed at the underlying cause. The incidence of NBTE in the general population is estimated based on autopsy series to range from 1.1% to 1.6%.^1^ It is usually associated with autoimmune diseases (systemic lupus erythematosus [SLE], antiphospholipid syndrome [APLS]), hypercoagulable states (APLS), or malignancy.^2^

## Summary figure

**Figure:**
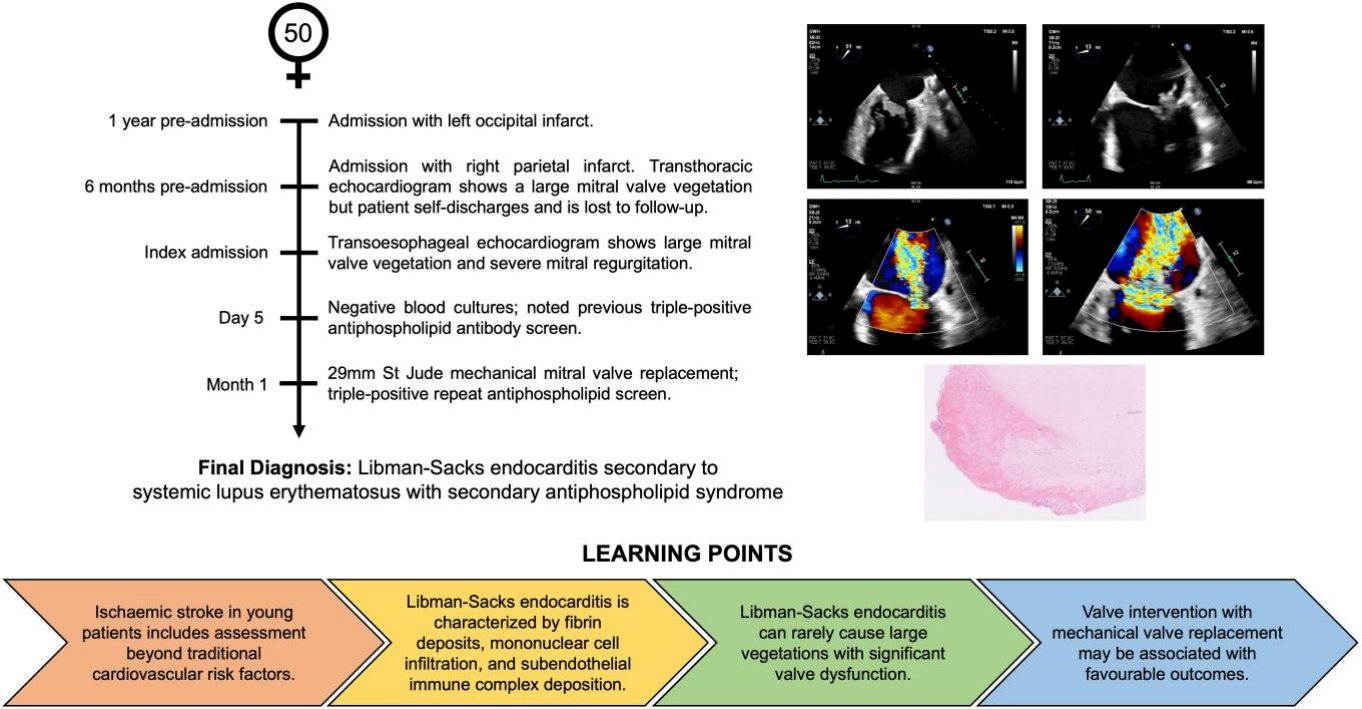


NBTE in the context of systemic autoimmune disease such as SLE is also referred to as Libman–Sacks endocarditis, after the physicians who first described it in four patients in 1924.^3^ Patients with SLE have only been systematically studied in small cohorts, where the estimated prevalence of Libman–Sacks endocarditis has been reported to be ∼10–40%.^2,4^ Most of these vegetations are reported to be small (<1 cm in diameter),^2^ not warranting action beyond treatment of the underlying autoimmune disorder and anticoagulant therapy.

Here, we present an atypical case of a patient with recurrent ischaemic stroke, who was discovered to have positive APLS antibodies and culture-negative endocarditis with a large mitral valve vegetation causing severe mitral regurgitation, with a histological diagnosis of Libman–Sacks endocarditis, ultimately warranting mitral valve replacement.

## Case presentation

**Figure 1:**
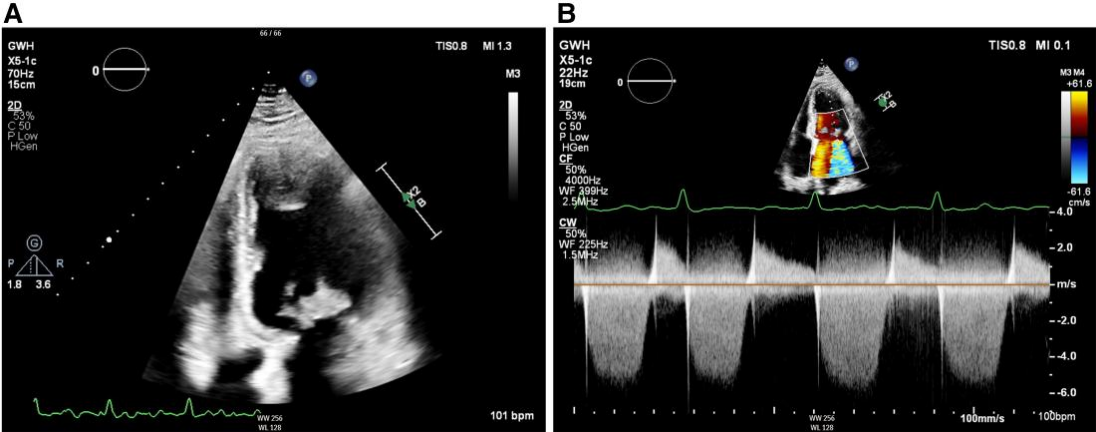
Transthoracic echocardiogram showing (*A*) a large mass associated with the mitral valve in apical four-chamber view; (*B*) continuous wave Doppler suggestive of severe mitral regurgitation.

A 50-year-old woman presented to her local hospital with left leg weakness. Imaging showed a right parietal stroke and an old occipital lobe stroke. Atrial fibrillation (AF) was newly diagnosed at the time. She had an iron deficiency anaemia (haemoglobin 77 g/L, normal range 115–165 g/L), for which an endoscopy showed erosive gastritis. Duplex ultrasound of the carotids showed no significant carotid artery disease. A transthoracic echocardiogram showed a 1.7 cm × 0.8 cm mass associated with the posterior mitral valve leaflet and moderate to severe mitral regurgitation (*Figure 1*). Blood cultures were negative, and she was afebrile with normal inflammatory markers. An inpatient transoesophageal echocardiogram (TOE) was planned, but she self-discharged against medical advice before this could be performed. She underwent outpatient computed tomographic (CT) imaging of the abdomen and pelvis for work-up of her anaemia, showing no malignancy, but was then lost to follow-up for 6 months, until persuaded to have her TOE.

**Figure 2:**
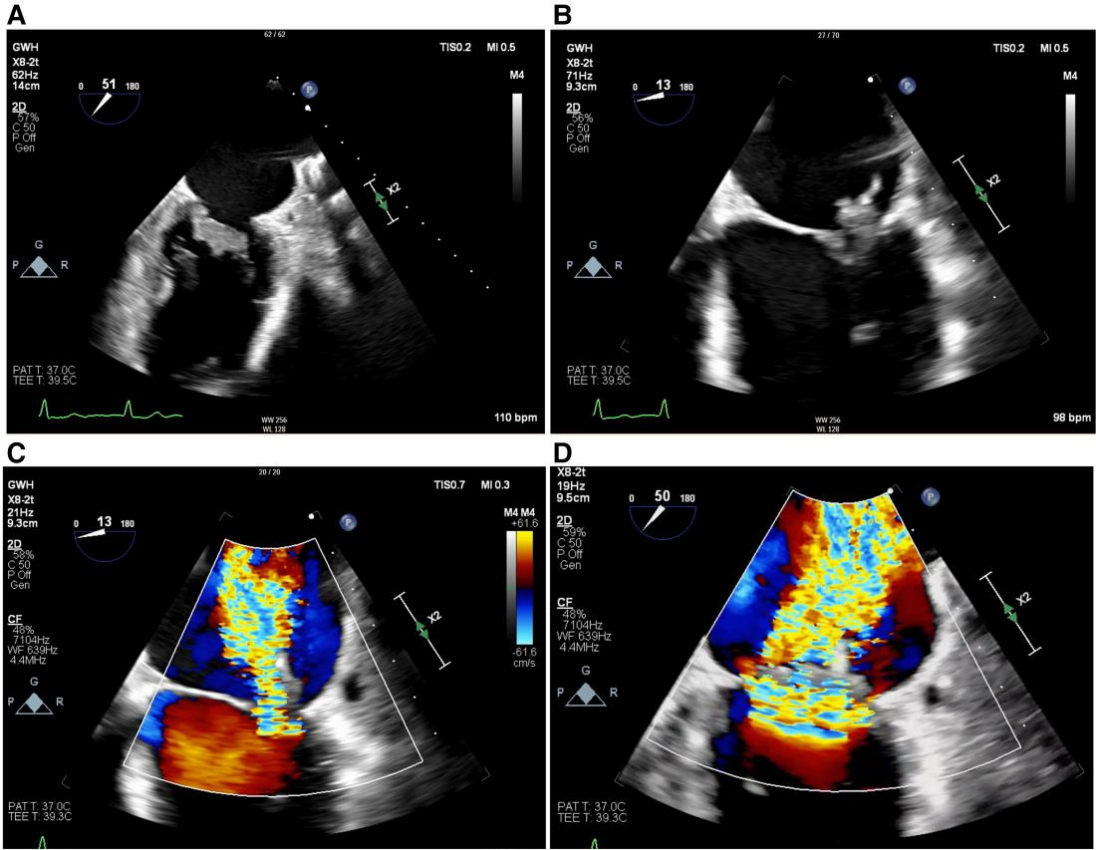
Transoesophageal echocardiogram: (*A*) four-chamber view showing a large mitral valve lesion; (*B*) two-chamber view showing a large mitral valve mass; (*C*) colour flow Doppler of the mitral valve in four-chamber view; and (*D*) in two-chamber view showing a broad jet of mitral regurgitation.

The TOE showed severe mixed mitral valve disease secondary to a large (2 cm × 3 cm) mass attached to the posterior mitral valve leaflet (*Figure 2*, Supplementary material online, *Video S1*). Severe mitral regurgitation was noted (estimated regurgitant orifice area 0.9 cm^2^, regurgitant volume 150 mL) secondary to valvular perforations (multiple jets noted). There was also moderate-severe mitral stenosis due to the obstructing effects of the vegetation itself (mean pressure gradient 10 mmHg, estimated mitral valve area 1.0 cm^2^). Biventricular systolic function was preserved (left ventricular ejection fraction 61%, tricuspid annular plane systolic excursion 18 mm), with no evidence of significant ventricular dilatation (left ventricular end-diastolic diameter 45 mm, right ventricular basal diameter 26 mm). She was directly admitted under the cardiology team from the TOE for monitoring and further investigation.

Her past medical history otherwise consisted of hypertension, asthma, and obesity. Ophthalmology review had previously demonstrated significant bilateral visual field defects and extensive retinal atrophy on optical computed tomography. She had one daughter and reported no history of miscarriage. She was an ex-smoker with a 15 pack-year history. Medications on admission included propranolol 80 mg once daily, amlodipine 10 mg once daily, ramipril 10 mg once daily, omeprazole 40 mg once daily, atorvastatin 80 mg once daily, clopidogrel 75 mg once daily (she self-discharged before this could be changed to anticoagulation), beclomethasone inhaler twice daily, and a salbutamol inhaler as required.

On this admission, she reported worsening dyspnoea on exertion over the last month. She was able to walk around her local shop but had not ventured beyond this recently. There was no history of weight loss, night sweats, fevers, rashes, or arthralgia. She reported a non-productive cough and two episodes of *per vaginal* bleeding; her last period had been over 1 year ago.

On examination, she was comfortable, without dyspnoea at rest. She was peripherally well-perfused, with an irregular pulse of 80 beats per minute. The jugular venous pressure could not be visualized. Auscultation revealed first and second heart sounds, with a holosystolic murmur radiating to the axilla; there were vesicular breath sounds with no crackles on lung auscultation. There were no splinter haemorrhages or peripheral stigmata of infective endocarditis. There was mild peripheral oedema to the lower shins bilaterally. The power in her limbs was normal. A 12-lead electrocardiogram showed rate-controlled AF.

Her inflammatory markers were unremarkable (C-reactive protein 6 mg/L, normal range 0–5 mg/L; white cell count 7.9 × 10^9^/L, normal range 4–10 × 10^9^/L). A normocytic anaemia (haemoglobin 100 g/L; mean cell volume 93.3 fl, normal range 83–101 fl) and thrombocytopaenia (platelet count 93 × 10^9^/L, normal range 150–400 × 10^9^/L) were noted. She had mildly impaired renal function (creatinine 99 µmol/L, normal range 45–84 µmol/L, estimated glomerular filtration rate 58 mL/min/1.73 m^2^) and a negative urine dip. Three sets of blood cultures were performed before antibiotic therapy was commenced to cover for infective endocarditis. Oral furosemide 40 mg once daily was commenced. Clopidogrel was changed to apixaban 5 mg twice daily.

Given her history of multiple ischaemic strokes, with a large vegetation, and normal inflammatory markers with previously negative cultures in an afebrile patient, the differentials at this stage included infective endocarditis from typical organisms, culture-negative infective endocarditis from fastidious organisms such as the ‘HACEK’ group (*Haemophilus*, *Aggregatibacter*, *Cardiobacterium*, *Eikenella*, *Kingella*), or intracellular organisms (*Bartonella*, *Coxiella*), or NBTE. Blood polymerase chain reaction (PCR) testing was requested to detect such organisms.

The admitting team carefully reviewed her previous investigations that included both autoimmune and thrombophilia screens. These showed negative anti-nuclear cytoplasmic antigen, anti-extractable nuclear antigen, anti-Ro, anti-La, anti-Jo1, and anti-Scl 70 antibodies. However, her APLS antibody profile was markedly positive. Lupus anticoagulant testing with a dilute Russell’s viper venom test showed a prolonged clotting time (ratio 4.03, normal 0.79–1.21); a coagulation profile performed with lupus-insensitive reagent was abnormal (prothrombin time 16.1 s, normal 8.7–13.8 s; activated partial thromboplastin time 34.3 s, normal 19–28.6 s). The anti-ß_2_-glycoprotein IgG titre was significantly raised (>160 units/mL, normal range 0–20.1 units/mL), as was the anti-cardiolipin IgG antibody titre (>160 units/mL, normal range 0–20.1 units/mL). Her anti-dsDNA antibodies were mildly raised (13 international units/mL, 0–10.1 international units/mL). Unfortunately, these results were not acted upon, as the patient was lost to follow-up when she previously self-discharged.

The rheumatologists were consulted for consideration of possible APLS and Libman–Sacks endocarditis. On their assessment, no features of systemic autoimmune disease were noted. Their impression was of triple-positive primary APLS and Libman–Sacks endocarditis. They advised repeat APLS antibody testing and transition of anticoagulation to warfarin, both of which were performed. They did not feel systemic immunosuppression was warranted. A referral to gynaecology was also made, with a transvaginal ultrasound showing a thickened endometrium and a normal-appearing uterus. An endometrial biopsy was performed, which showed a benign polyp only. In the meantime, she remained afebrile with negative blood cultures and no culture-negative organisms on blood PCR. Her antibiotics were stopped.

Her case was discussed in a joint cardiothoracic-cardiology multidisciplinary meeting, and she was accepted for mitral valve replacement. A coronary angiogram showed unobstructed coronary arteries.

**Figure 3:**
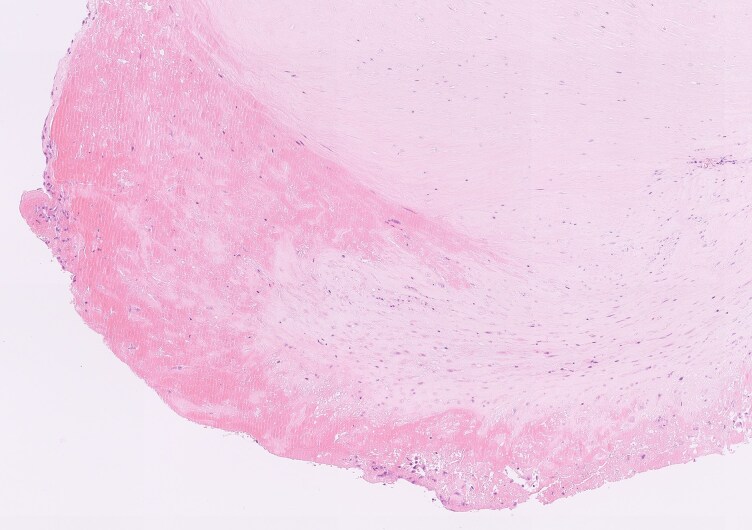
Haematoxylin and eosin (H&E) stain of valve leaflet with non-bacterial thrombotic endocarditis. The valve leaflet shows bland featureless eosinophilic mass of fibrinoid material, with scattered monocytes and occasional neutrophils, adjacent to fibrous tissue (H&E, 150×).

She was transferred to a surgical centre and underwent mitral valve replacement with low molecular weight heparin bridging, on advice from the haematologists. Intra-operatively, the mitral valve was grossly pathological, with a large vegetation and destruction of the valve leaflets. A 29 mm St Jude mechanical mitral valve was implanted, with good function (mean gradient 3.4 mmHg, heart rate 76 beats per minute) and no paravalvular leak on an on-table TOE. Post-operative histology showed features consistent with NBTE, with predominant fibrinoid material and scattered leucocytes (*Figure 3*), confirming Libman–Sacks endocarditis. Tissue culture did not yield any infective organisms. A repeat APLS antibody profile showed persistent triple positivity, confirming APLS. She was discharged 26 days post-operation, after an uneventful recovery.

**Figure 4:**
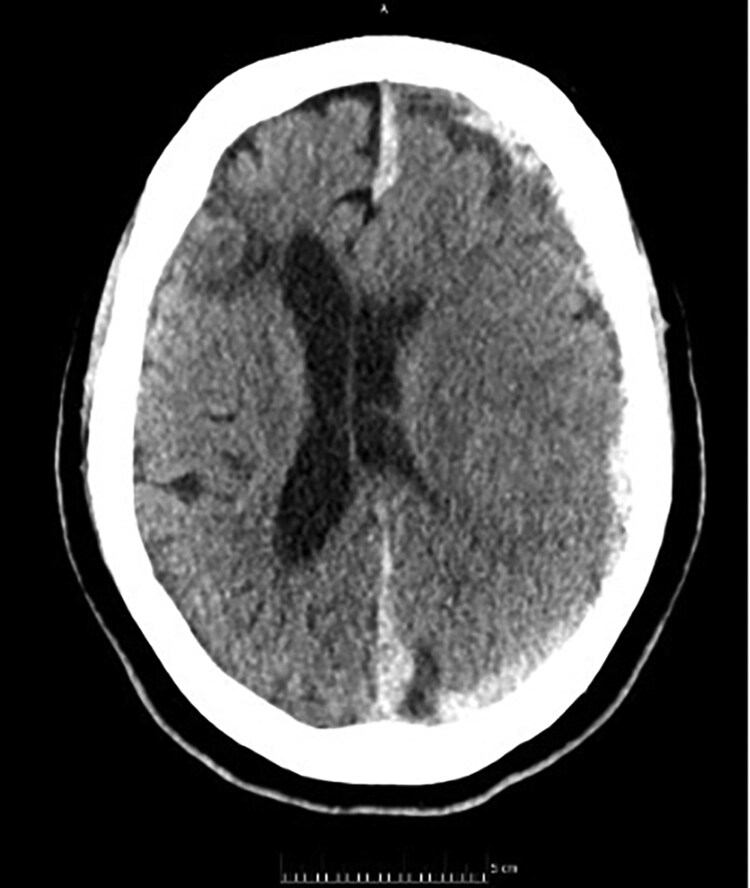
Computed tomographic imaging of the brain on presentation with dysphasia and confusion 7 months post-operatively, showing a left subdural haematoma with mass effect (midline deviation).

Unfortunately, she presented to hospital 7 months post-operation with sudden onset confusion, dysarthria, and ataxia. Her international normalized ratio 3 days prior was 8.9 (target 2.5–3.5). A CT head showed a large left subdural haematoma, 9 mm in depth with mass effect (*Figure 4*). She was transferred to a neurosurgical centre and underwent craniotomy and evacuation of the subdural haematoma. The immediate post-operative course was complicated by partial seizures and progression of dysphasia. A repeat CT head on post-operation day 2 showed a new left frontal intraparenchymal haemorrhage, with some mass effect. This was treated conservatively, but anticoagulation was withheld by the neurosurgical team due to concerns over bleeding risk. Prophylactic dose low molecular weight heparin (dalteparin 5000 units) was commenced at post-operative day 7. A further CT head at post-operative day 10 showed relatively stable appearances of the haemorrhage, but an increase in vasogenic oedema. Treatment dose low-molecular weight heparin (dalteparin 18 000 units) was recommenced at this point. A transthoracic echocardiogram at this time showed a well-seated mitral valve prosthesis with no rocking or regurgitation, and a mean gradient of 7.3 mmHg (heart rate 78 beats per minute). At post-operative day 17, warfarin loading (5 mg per day) was commenced.

After rehabilitation and discharge, she was reviewed in the outpatient clinic by the rheumatologists, who repeated her autoantibody profile. Her APLS antibody panel was again positive for all three APLS antibodies. Furthermore, anti-nuclear antibody testing was positive (1:80, with a speckled appearance), and her anti-dsDNA was raised. A urine dip was negative. On this basis, the rheumatology team commenced hydroxychloroquine for a diagnosis of SLE [based on the European League Against Rheumatism (EULAR) and American College of Rheumatology (ACR) criteria: positive ANA, associated antibodies (anti-dsDNA), thrombocytopaenia, APLS antibodies]. A transthoracic echocardiogram conducted as part of cardiac surgical follow-up showed a mean gradient of 3.0 mmHg (heart rate 75 beats per minute).

Finally, she suffered from a further brief hospital admission ∼1 year after index presentation with fevers, with blood cultures positive for *Escherichia coli*. A TOE showed no evidence of infective endocarditis of the mitral valve prosthesis or the native valves. The valve prosthesis was well-seated and opened well (mean gradient 4.8 mmHg; heart rate 110 beats per minute) with no significant regurgitation. Two weeks of antibiotic therapy resulted in clear clinical improvement, with resolution of fever, improvement in inflammatory markers, and negative repeat blood cultures, and thus she was discharged with outpatient follow-up. At last follow-up, 1 year after her index presentation, she was stable, with no cardiovascular symptoms of note, and no evidence of further thrombo-embolic phenomena.

## Discussion

Here, we describe a case of a young patient with multiple ischaemic strokes, who was ultimately diagnosed with SLE with APLS, with associated Libman–Sacks endocarditis causing severe mitral valvular regurgitation and valve destruction requiring valve replacement (*Table 1*). Although Libman–Sacks endocarditis is a recognized association with SLE, the large size of the vegetation (3 cm), its significant effects on valvular function necessitating surgical intervention, and first presentation of SLE with NBTE, are not typical for this diagnosis.

**Table 1:** Timeline of events

Time	Event
**1 year prior**	Admission with headache and visual blurring; imaging showing left occipital infarct
**6 months prior**	Admission with left leg weakness and imaging showing right parietal ischaemic stroke. In atrial fibrillation at the time, with transthoracic echocardiogram showing moderate to severe mitral regurgitation with a mitral valve mass. Self-discharged before formal anticoagulation could be commenced, and before transoesophageal echocardiogram could be performed
**2 months prior**	Positive antiphospholipid antibody profile testing, with markedly elevated lupus anticoagulant, anti-ß_2_-glycoprotein IgG, and anticardiolipin IgG
**Index admission (Day 0)**	Outpatient transoesophageal echocardiogram showing large mitral valve vegetation with severe mitral regurgitation; admitted to cardiology ward and commenced on apixaban for atrial fibrillation
**Day 2**	Review of previous results shows positive antiphospholipid screen; anticoagulation changed to warfarin
**Day 5**	Blood cultures negative
**Month 1**	Mitral valve replacement with a 29 mm St Jude mechanical mitral valve; repeat antiphospholipid profile (performed earlier in index admission) positive
**Month 2**	Discharged from hospital
**Month 8**	Presentation with sudden onset expressive dysphasia, confusion, ataxia, with CT head showing a large left subdural haematoma with mass effect. Transferred to neurosurgical centre and underwent craniotomy and evacuation of subdural haematoma
**Month 9**	After neurosurgery, warfarin resumed; transoesophageal echocardiogram showing well-seated mechanical mitral valve with good function
**Month 10**	Seen in outpatient rheumatology clinic; autoimmune screen repeated with positive anti-nuclear antigen (1:80), anti-dsDNA antibodies, and antiphospholipid antibodies. Commenced on hydroxychloroquine
**Month 12**	Further admission with fevers and *Escherichia coli* bacteraemia; transoesophageal echocardiogram showed stable mechanical mitral valve prosthesis, with no evidence of prosthetic or native valve endocarditis. No thrombo-embolic phenomena. Bacteraemia resolved with intravenous antibiotics

Systematic reports of Libman–Sacks endocarditis are limited, with prevalence data from imaging studies and small histopathological series (since lesions are not always resected). Systematic echocardiographic studies in patients with SLE report that valvular vegetations are present in ∼10% of patients with SLE, though reports vary and are limited by small cohorts.^4^ There may be an association with antiphospholipid antibody positivity.^5^ Vegetations have a predilection for the left-sided cardiac valves (hypothesized to be secondary to the greater shear stresses experienced by these valves, potentially predisposing to endothelial injury), forming predominantly at the tips.^2^ Most lesions are small (generally <1 cm in diameter).^2^ Vegetations generally consist of fibrin deposits and mononuclear cell infiltration, as seen in this case.^6^ In patients with antiphospholipid antibodies, immunohistochemical examination demonstrates subendothelial immune complex deposition, highlighting a potential pathogenic role of antiphospholipid antibodies in this process.^7^

There are no established guidelines for management of Libman–Sacks endocarditis. The 2023 European Society for Cardiology Guidelines on management of endocarditis state that treatment of the underlying cause should be undertaken, and anticoagulation with heparin or vitamin K antagonists considered in all patients. It suggests that while the role of surgery is controversial, it should be considered in patients with severe valvular dysfunction or large vegetations.^1^ There have been reported cases of regression of Libman–Sacks endocarditis after treatment with immunosuppression, in one case with steroids and hydroxychloroquine with aspirin,^8^ and in another case with prednisolone, cyclosporin, and hydroxychloroquine where there was reduction of mitral regurgitation from severe to mild;^9^ in both cases, Libman–Sacks endocarditis was found in the context of SLE with multisystem involvement requiring immunosuppression, and consisted of valvular thickening^9^ or small vegetations^8^ only. In a separate case of Libman–Sacks endocarditis on a background of SLE being managed with immunosuppression, transcatheter edge-to-edge repair was successfully performed, but again the appearances of the mitral valve were predominantly of leaflet thickening with small vegetations.^10^ In our case, it was felt that valve replacement was indicated due to severe valvular dysfunction with the presence of a large vegetation. Valve replacement, usually undertaken with mechanical prostheses, appears to be associated with good outcomes;^11,12^ there are theoretical concerns with early valve degradation and disease recurrence in bioprosthetic valves,^13^ though evidence of superiority of either modality has not been demonstrated.

Our case highlights the complexity in diagnosis and management of the combination of SLE, APLS, and Libman–Sacks endocarditis. Firstly, diagnosis requires multidisciplinary co-ordination between cardiologists, rheumatologists, and haematologists. Libman–Sacks endocarditis as a first presenting feature of SLE is rarely reported.^14^ When valvular vegetations are the first detected abnormality, the primary differential is usually of infective endocarditis given the lack of known history of autoimmune disease, which may lead to delays in diagnosis and treatment (particularly as treatment may involve immunosuppression, in contrast with antibiotic therapy for infective endocarditis). Secondly, careful evaluation of the cause of NBTE once this diagnosis is established is also required, as done in this case (CT, endoscopy, endometrial biopsy), as malignancy is another important association that must be ruled out. Thirdly, management of anticoagulation in patients with SLE, APLS, and Libman–Sacks endocarditis can be challenging and may require haematology input. Testing for APLS requires temporary cessation of anticoagulant therapy. If operative management is pursued, further careful titration of anticoagulation is required in the peri-operative period to ensure sufficient thromboprophylaxis while allowing adequate intra-operative haemostasis. Although APLS is characteristically a syndrome of thrombosis, the use of vitamin K antagonists and other predisposing factors (e.g. thrombocytopaenia) can lead to elevated risk of bleeding events,^15^ as seen in our case. Despite the widespread use of direct-acting oral anticoagulants for other thrombotic indications, which have been shown to be safer from a bleeding perspective in other contexts, trials have failed to demonstrate safety or efficacy of these agents over vitamin K antagonists in APLS.^16,17^ Finally, our case highlights the long-term challenges of managing Libman–Sacks endocarditis after valve replacement alongside APLS and SLE. Careful surveillance of valve prostheses is necessary for prompt detection of complications, as highlighted by the two re-admissions. During the patient’s first re-admission with a subdural haematoma, anticoagulant therapy was withheld due to the presence of intracranial bleeding, with a complicated post-operative course. The repeat transthoracic echocardiogram performed at recommencement of anticoagulation showed an elevated mean gradient (7.3 mmHg, compared with 3.4 mmHg on the post-operative echocardiogram). While increases in mitral valve prosthesis gradients can be multifactorial, in this case, the two primary differentials of concern would be recurrent Libman–Sacks endocarditis or prosthetic valve thrombus. Recurrent Libman–Sacks endocarditis has been reported in bioprostheses, but, to the authors’ knowledge, has not been reported in mechanical prostheses, and would be highly uncharacteristic. Mechanical valve-associated thrombosis would be a more likely explanation, given the interruption of anticoagulation, known APLS, and the (fortunate) improvement in gradients at follow-up echocardiography after reinstitution of anticoagulant therapy. In this case, further imaging with TOE or CT may have been helpful to identify the aetiology of the elevated gradients. Alternative anticoagulation management strategies (e.g. early re-introduction of anticoagulation with heparin bridging, allowing close monitoring, titration, and further interruption if needed) might have been helpful, but the patient was under the care of a non-cardiac team at the time, in an external hospital, highlighting the value of collaborative care in such cases. The second re-admission highlights the challenges of balancing systemic immunosuppressive therapy against enhanced vulnerability for infective endocarditis (given underlying valvular abnormalities due to previous episodes of Libman–Sacks endocarditis and presence of a valvular prosthesis), requiring close collaboration between cardiologists and rheumatologists. In this case, further investigation with a TOE was performed early in the patient’s admission. Although an elevated gradient was again documented, the TOE showed no evidence of vegetations. The transmitral gradient is not only affected by valve area (i.e. presence of stenosis), but by factors that influence the mitral flow rate: here, the fact that the patient was tachycardic at the time of TOE. In this case, given clear clinical improvement with intravenous antibiotic therapy and no suspicious findings on TOE, the patient was discharged without further investigation, but in cases where the diagnosis remains uncertain, further imaging (e.g. with positron-emission tomography CT) should be considered.

A final important aspect of this case is the history of recurrent stroke in a young patient. Although ischaemic stroke is much more common in elderly patients, it is increasingly recognized in younger populations. Much of this may be secondary to poorer control of traditional cardiovascular risk factors, with rising rates of dyslipidaemia, hypertension, diabetes mellitus, smoking, and obesity.^18^ However, young patients (generally under 50 years old) with ischaemic stroke also have a higher prevalence of atypical underlying risk factors and causes. These might include: contraception use and pregnancy, inherited thrombophilias or acquired hypercoagulable states (Factor V Leiden, protein C and S deficiency, antithrombin III deficiency, APLS, SLE, sickle cell disease, malignancy), carotid/vertebral artery dissection, arteriovenous malformations, vasculopathy and vasculitides, cardioembolism, and patent foramina ovale.^18^ Thus, in young patients with ischaemic stroke, specific work-up may be indicated: this in the first instance usually consists of a dedicated history and physical examination, thrombophilia screen, cerebrovascular imaging, and a transthoracic echocardiogram. In this case, the recurrent ischaemic strokes were likely caused either by a hypercoagulable state secondary to triple-positive APLS or embolism from the mitral valve vegetation.

## Conclusion

In young patients with ischaemic stroke, atypical causes should be considered. The presence of culture-negative endocarditis should prompt the search for fastidious organisms and, particularly when associated with embolic phenomena, causes of NBTE (autoimmune disorders, coagulopathy, malignancy). Although Libman–Sacks endocarditis typically manifests as small vegetations managed with treatment of the underlying condition and anticoagulation, it can result in large vegetations causing significant valve dysfunction requiring intervention, which can be performed safely with good results.

## Supplementary Material

ytaf647_Supplementary_Data

## Data Availability

No new data were generated or analysed in support of this research.

## Lead author biography

Dr Tang is currently a clinical research fellow at the Radcliffe Department of Medicine, University of Oxford, pursuing a DPhil in Clinical Medicine funded by the British Heart Foundation on atrial cardiomyopathy, left atrial blood flow, and stroke. He completed his medical degree at the University of Oxford, including a period of time at the UCLA Cardiac Arrhythmia Center as a visiting research student, and has since undergone training in internal medicine and cardiology in the Oxford region. He is undergoing his current research post as part of time taken out of his clinical training as a cardiology registrar in the Oxford deanery, with a long-term aim of a career in cardiac electrophysiology.

## Primary specialties involved other than cardiology

Cardiothoracic surgery, rheumatology, haematology, stroke medicine, and haematology.
